# Association Between Time to Antiretroviral Therapy and Loss to Care Among Newly Diagnosed Rwandan People Living with Human Immunodeficiency Virus

**DOI:** 10.1089/aid.2022.0023

**Published:** 2023-05-05

**Authors:** Gad Murenzi, Hae-Young Kim, Qiuhu Shi, Benjamin Muhoza, Athanase Munyaneza, Gallican Kubwimana, Eric Remera, Sabin Nsanzimana, Marcel Yotebieng, Denis Nash, Kathryn Anastos, Jonathan Ross

**Affiliations:** ^1^ Rwanda Military Hospital, Kigali, Rwanda.; ^2^ Research for Development (RD Rwanda), Kigali, Rwanda.; ^3^ New York Medical College, Valhalla, New York, USA.; ^4^ Rwanda Biomedical Center, Kigali, Rwanda.; ^5^ Albert Einstein College of Medicine, Bronx, New York, USA.; ^6^ Institute for Implementation Science in Population Health, City University of New York, New York, New York, USA.; ^7^ School of Public Health, City University of New York, New York, New York, USA.

**Keywords:** ART initiation, HIV, loss to care, viral suppression, retention in care, Treat All

## Abstract

Despite improved clinical outcomes of initiating antiretroviral therapy (ART) soon after diagnosis, conflicting evidence exists regarding the impact of same-day ART initiation on subsequent clinical outcomes. We aimed to characterize the associations of time to ART initiation with loss to care and viral suppression in a cohort of newly diagnosed people living with HIV (PLHIV) entering care after Rwanda implemented a national “Treat All” policy. We conducted a secondary analysis of routinely collected data of adult PLHIV enrolling in HIV care at 10 health facilities in Kigali, Rwanda. Time from enrollment to ART initiation was categorized as same day, 1–7 days, or >7 days. We examined associations between time to ART and loss to care (>120 days since last health facility visit) using Cox proportional hazards models, and between time to ART and viral suppression using logistic regression. Of 2,524 patients included in this analysis, 1,452 (57.5%) were women and the median age was 32 (interquartile range: 26–39). Loss to care was more frequent among patients who initiated ART on the same day (15.9%), compared with those initiating ART 1–7 days (12.3%) or >7 days (10.1%), *p* < .001. In multivariable analyses, same-day ART initiation was associated with a greater hazard of loss to care compared with initiating >7 days after enrollment (adjusted hazard ratio 1.39, 95% confidence interval: 1.04–1.85). A total of 1,698 (67.3%) had available data on viral load measured within 455 days after enrollment. Of these, 1,476 (87%) were virally suppressed. A higher proportion of patients initiating ART on the same day were virally suppressed (89%) compared with those initiating 1–7 days (84%) or >7 days (88%) after enrollment. This association was not statistically significant. Our findings suggest that ensuring adequate, early support for PLHIV initiating ART rapidly may be important to improve retention in care for newly diagnosed PLHIV in the era of Treat All.

## Introduction

Initiating antiretroviral therapy (ART) soon after diagnosis reduces HIV-related morbidity and mortality as well as onward HIV transmission.^1,2^ The World Health Organization's (WHO) “Treat All” guidelines thus recommend ART for all people living with HIV (PLHIV) within 7 days after diagnosis with same-day ART initiation for those who are ready to start.^3^ Treat All policies, which have been adopted by nearly all countries globally, have the potential to fast track achieving the 2025 UNAIDS 95-95-95 targets.^4^

Despite improved clinical outcomes of initiating ART soon after diagnosis, conflicting evidence exists regarding the impact of same-day ART initiation on loss to care and viral suppression. A meta-analysis by Ford et al., assessed findings from three clinical trials, which indicated that same-day ART initiation increased viral suppression and retention in care at 12 months but the latter showed a borderline statistical significance. The same meta-analysis also reviewed findings from three observational studies, which showed that rapid ART initiation was associated with a trend toward an increased risk of being lost to care at 6 months. No association was found with reduced mortality or reduced loss to care at 12 months.^5^

Another study carried out in Botswana indicated no difference in loss to care among those who initiated ART on the same day compared with those who initiated ART before the “Treat All” policy.^6^ Subsequent observational studies carried out in sub-Saharan Africa (SSA) have described increased loss to care among patients initiating ART on the same day as diagnosis, compared with those who initiated it later.^7–9^

In contrast, findings of improved retention in care at 12 months and viral suppression among those who initiated ART on the same day compared with standard of care (ART initiation 3 weeks after HIV diagnosis) were observed in a study carried out in Haiti.^10^ In addition, a study carried out in the United States showed no difference in loss to care between same-day ART initiation and routine care but viral suppression was better among same-day initiation patients.^11^

Although an increasing number of HIV programs in SSA are implementing rapid ART initiation protocols, there remain unanswered questions about the impact of such approaches on subsequent clinical outcomes. First, most studies have not directly compared loss to care among patients initiating ART on the day as enrollment with those initiating immediately afterward (i.e., 1–7 days). Second, few studies have examined the association between time to ART initiation and subsequent viral suppression.

Rwanda implemented “Treat All” with rapid ART initiation nationally in all health facilities in July 2016. At present, >90% of PLHIV in Rwanda are on ART and Rwanda has achieved the previous 90-90-90 goals of the UNAIDS.^12^ Newly diagnosed people with HIV are increasingly initiated on ART soon after diagnosis.^13,14^ Despite the widespread rollout of Treat All and rapid ART initiation in Rwanda, the impact on loss to care and viral suppression has not yet been studied. We therefore aimed to characterize the associations of time to ART initiation with loss to care and viral suppression in a cohort of PLHIV entering care after Rwanda implemented “Treat All.”

## Materials and Methods

### Design, setting and population

This was an analysis of routinely collected longitudinal data on PLHIV enrolling in HIV care at 10 health facilities located in Kigali, Rwanda that collectively serve ∼36,000 PLHIV and which collaborate with the Central Africa International epidemiology Databases to Evaluate AIDS (CA-IeDEA) consortium.^15^ CA-IeDEA methods have been previously described.^16^ Eight of the sites are public health centers, one is a private HIV clinic, and one is a public referral hospital [Rwanda Military Hospital (RMH)]. Kigali, the capital of and largest city in Rwanda, has a population of ∼1 million people with an adult HIV prevalence of 4.3%.^12^

Rwanda guidelines, in place during the study period, recommended that all newly diagnosed PLHIV be initiated on ART within 7 days of diagnosis, and on the same day if possible depending on the psychosocial readiness of the patient. Those initiated on ART are scheduled for monthly pharmacy appointments and quarterly clinical assessments for the first 18 months after initiation. Stable PLHIV—adults on ART for ≥18 months and with two consecutive suppressed viral loads—come for quarterly pharmacy appointments and semi-annual clinical assessments. Most patients (80%–90%) in our study population were prescribed the Tenofovir/Lamivudine/Efavirenz first-line regimen. Current guidelines also recommend viral load monitoring at 6 months after ART initiation and then yearly thereafter.^17^

**FIG. 1.**
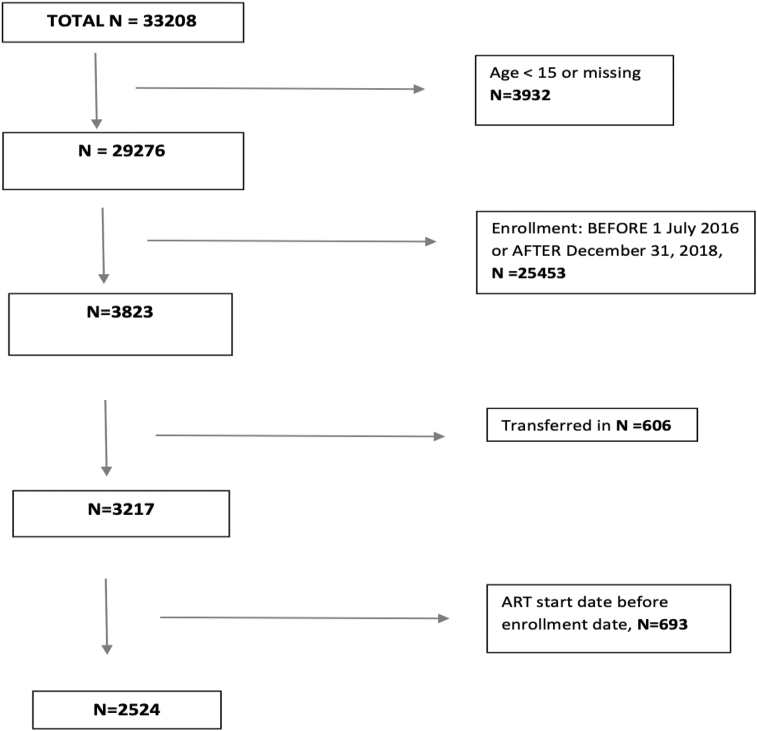
Flow chart of inclusion and exclusion for analysis.

For this analysis, we included all newly enrolled PLHIV receiving care at the IeDEA collaborating health facilities, who were ≥15 years, and enrolled in care between July 1, 2016 and December 31, 2018. We excluded patients who were known to have transferred in from outside health facilities and those with evidence of ART initiation before enrollment in care at study health facilities (Fig. 1).

### Data collection

In study facilities, routine clinical data from the medical records of PLHIV are routinely input into the Open Medical Record System (OpenMRS), an electronic medical record system. Data are de-identified and extracted every 3–6 months into a research database maintained by CA-IeDEA.^18^ Data for this analysis were extracted into the study database on September 25, 2019.

### Definition of variables

The primary outcome, loss to care, was defined as >120 days between the last health facility visit (pharmacy or clinical appointment) and the database closure, to account for the possibility that some patients may be late to their appointments, which are scheduled 90 days apart. Outcomes among those lost to care (i.e. deaths, care status, ART status) were not available. Viral suppression (<200 copies/mL, as defined in the Rwanda national guidelines), measured on the first viral load performed within 455 days after enrollment, was the secondary outcome variable.

The primary predictor was time from enrollment in HIV care to ART initiation, categorized as same day, 1–7 days, and >7 days after enrollment in care. Other independent variables included age (15–23, 24–35, and >35 years), gender (female, male), enrollment period (July 2016 to June 2017, July 2017 to June 2018 and July 2018 to December 2018), referral source [voluntary counseling and testing (VCT), prevention of mother-to-child transmission (PMTCT), other], body mass index (BMI) (<18.5, 18.5–24, and >24 kg/m^2^), CD4 count (<200, 200–349, 350–499, and ≥500 cells/μL), and WHO stage (stage I/stage II, and stage III or stage IV).

### Analysis

Demographic and clinical characteristics by time to ART initiation groups were compared using the chi-square test for categorical variables and Kruskal–Wallis test for continuous variables. Patients were followed from the date of enrollment until date of their last clinic visit (if they had one of the following events: lost to care, documented transfer, or documented death) or for 455 days after enrollment. Survival probability of loss to care was determined by Kaplan–Meier analysis, and differences between time to ART initiation groups were compared by the log-rank test. Cox proportional hazard regression models were used to evaluate the association between characteristics at enrollment and loss to care among participants who initiated ART. Logistic regression models were used to evaluate the association between characteristics at enrollment and viral suppression among all patients who initiated ART and with available viral load data. For multivariable Cox and logistic regression models, we included the primary predictor variable of time to ART as well as covariates that were statistically significant in univariate analyses. Missing category was included for the analysis for CD4 count, referral source, BMI, and WHO stage. All analyses were performed with SAS statistical software (SAS Institute, Cary, NC, USA). Values of *p* < .05 were considered statistically significant.

### Ethical consideration

The CA-IeDEA study protocol was reviewed and approved by both the Albert Einstein College of Medicine's institutional review board and the Rwanda National Ethics Committee. Informed consent was waived for the study as it involves only secondary, de-identified data extracted from patient medical records; however, CA-IeDEA data entry staff sign annual confidentiality agreements.

## Results

Of the 2,524 patients included in this analysis, 1,452 (57.5%) were women, and the median age was 32 [interquartile range (IQR): 26–39]. Most patients (41%) enrolled between July 2017 and June 2018 and most (75.9%) were referred from VCT. At baseline, median BMI was 22 kg/m^2^ (IQR: 19–24), the majority of patients (64.1%) were classified as WHO stage I and the median CD4 cell count was 415 cells/mm^3^ (IQR: 246–619), with 38.7% missing CD4 count. Overall, 127 patients (5%) did not initiate ART during the study period. Compared with those initiating later, patients who initiated ART on the same day as enrollment were younger, more likely to be women, and more likely to enroll through PMTCT programs (*p* < .001 for all) (Table 1).

**Table 1.** Demographic and Clinical Characteristics of 2,524 Adult People Living with HIV Enrolling in Care in 10 Health Facilities in Kigali, Rwanda

Variable	Total (*N* = 2,524)	Time to ART				*p*
		Same day (*n* = 860)	1–7 days (*n* = 756)	>7 days (*n* = 781)	Did not start	
Sex						
Female	1,452 (57.5)	568 (66.0)	399 (52.8)	399 (51.1)	86 (67.7)	<.001
Male	1,072 (42.5)	292 (34.0)	357 (47.2)	382 (48.9)	41 (32.3)	
Median age (IQR)	32 (26–39)	30 (25–37)	34(28–42)	33 (27–39)	32 (24–38)	<.001
Age (years)						
15–23	346 (13.7)	149 (17.3)	80 (10.6)	90 (11.5)	27 (21.3)	<.001
24–35	1,252 (49.6)	457 (53.1)	344 (45.5)	395 (50.6)	56 (44.1)	
>35	926 (36.7)	254 (29.5)	332 (43.9)	296 (37.9)	44 (34.6)	
Enrollment period						
July 2016 to June 2017	984 (39.0)	242 (28.1)	281 (37.2)	406 (52.0)	55 (43.3)	<.001
July 2017 to June 2018	1,034 (41.0)	391 (45.5)	305 (40.3)	285 (36.5)	53 (41.7)	
July 2018 to December 2018	506 (20.0)	227 (26.4)	170 (22.5)	90 (11.5)	19 (15.0)	
Referral source						
VCT	1,916 (75.9)	560 (65.1)	649 (85.8)	635 (81.3)	72 (56.7)	<.001
PMTCT	267 (10.6)	161 (18.7)	53 (7.0)	48 (6.1)	5 (3.9)	
Missing	341 (13.5)	139 (16.2)	54 (7.1)	98 (12.5)	50 (39.4)	
BMI (kg/m^2^)						
<18.5	286 (11.3)	72 (8.4)	83 (11.0)	112 (14.3)	19 (15.0)	<.001
18.5–24	1,175 (46.6)	363 (42.2)	387 (51.2)	397 (50.8)	28 (22.0)	
>24	521 (20.6)	188 (21.9)	197 (26.1)	127 (16.3)	9 (7.1)	
Missing	542 (21.5)	237 (27.6)	89 (11.8)	145 (18.6)	71 (55.9)	
WHO stage						
Stage I	1,617 (64.1)	546 (63.5)	530 (70.1)	496 (63.5)	45 (35.4)	<.001
Stage II	295 (11.7)	73 (8.5)	119 (15.7)	96 (12.3)	7 (5.5)	
Stage III and IV	126 (5.0)	27 (3.1)	32 (4.2)	62 (7.9)	5 (3.9)	
Missing	486 (19.3)	214 (24.9)	75 (9.9)	127 (16.3)	70 (55.1)	
CD4 count (cells/μL)						
≥500	580 (23.0)	187 (21.7)	156 (20.6)	222 (28.4)	15 (11.8)	<.001
350–499	341 (13.5)	110 (12.8)	104 (13.8)	122 (15.6)	5 (3.9)	
200–349	345 (13.7)	88 (10.2)	139 (18.4)	111 (14.2)	7 (5.5)	
<200	282 (11.2)	54 (6.3)	108 (14.3)	114 (14.6)	6 (4.7)	
Missing	976 (38.7)	421 (49.0)	249 (32.9)	212 (27.1)	94 (74.0)	
LTFU defined by >120 days						
No	2,163 (85.7)	723 (84.1)	663 (87.7)	702 (89.9)	75 (59.1)	<.001
Yes	361 (14.3)	137 (15.9)	93 (12.3)	79 (10.1)	52 (40.9)	
Time to LTFU (days)						
Median (IQR)	455 (337–455)	455 (272–455)	455 (415–455)	455 (455–455)	111 (28–455)	<.001
Time to LTFU (days, only LTFU = Yes)						
Median (IQR)	181 (80–298)	161 (71–276)	209 (100–320)	237 (171–344)	96 (17–131)	<.001
First VL suppression (<200)						
VL ≥200	222 (8.8)	62 (7.2)	92 (12.2)	68 (8.7)		<.001
VL <200	1,476 (58.5)	505 (58.7)	489 (64.7)	482 (61.7)		
Missing	826 (32.7)	293 (34.1)	175 (23.1)	231 (29.6)	127 (100)	

ART, antiretroviral therapy; BMI, body mass index; CD4, cluster of differentiation 4; IQR, interquartile range; LTFU, lost to follow-up; PMTCT, prevention of mother-to-child transmission; VCT, voluntary counseling and testing; VL, viral load; WHO, World Health Organization.

**FIG. 2.**
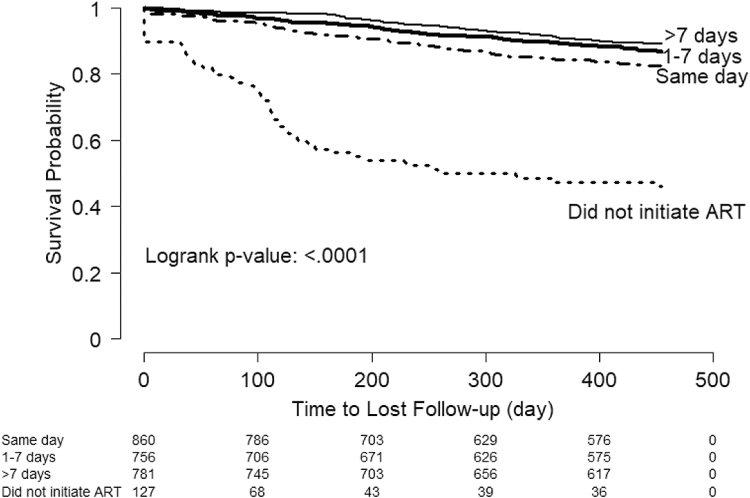
Kaplan–Meier curve for time to loss to care, by time from enrollment to ART initiation. ART, antiretroviral therapy.

Overall, 14.3% of participants were lost to care, including 6.4% with no visits after ART initiation and 93.6% with >1 visit after ART initiation. This outcome was more likely among patients who initiated ART on the same day (15.9%), compared with those initiating ART 1–7 days (12.3%) or >7 days after enrollment (10.1%) (*p* < .001). Among those who did not start ART, 40.9% were lost to care (Fig. 2).

In multivariable analyses, same-day ART initiation was associated with a higher hazard of loss to care compared with initiating ART >7 days after enrollment [adjusted hazard ratio (aHR) 1.39, 95% confidence interval (CI): 1.04–1.85] (Table 2). Other factors associated with loss to care included missing CD4 cell count (vs. >500 cells/mm^3^, aHR = 1.93, 95% CI = 1.40–2.67), enrolling in care during July 2018 to December 2018 (vs. July 2016 to June 2017, aHR = 1.78, 95% CI = 1.30–2.44), and age 15–23 years (vs. >35 years, aHR = 2.51, 95% CI = 1.82–3.46).

**Table 2.** Associations of Demographic and Clinical Characteristics at Enrollment and Loss to Care (*N* = 2,397)

Variable	*n* (%)	HR (95% CI)	aHR (95% CI)
Days ART initiation			
Same day (reference)	860 (35.9)		
1–7 days	756 (31.5)	0.71 (0.55–0.93)	0.81 (0.61–1.06)
>7 days	781 (32.6)	0.58 (0.44–0.76)	0.72 (0.54–0.96)
Sex			
Female (reference)	1,366 (57.0)		
Male	1,031 (43.0)	1.08 (0.86–1.35)	
Age (years)			
15–23	319 (13.3)	2.59 (1.89–3.55)	2.51 (1.82–3.46)
24–35	1,196 (49.9)	1.33 (1.02–1.73)	1.30 (1.00–1.70)
>35 (reference)	882 (36.8)		
CD4 count (cells/mm^3^)			
≥500 (reference)	565 (23.6)		
350–499	336 (14.0)	1.16 (0.77–1.76)	1.25 (0.83–1.90)
200–349	338 (14.1)	1.12 (0.74–1.70)	1.25 (0.83–1.91)
<200	276 (11.5)	0.69 (0.41–1.17)	0.87 (0.51–1.49)
Missing	882 (36.8)	2.12 (1.56–2.89)	1.93 (1.40–2.67)
Enrollment period			
July 2016 to June 2017 (reference)	929 (38.8)		
July 2017 to June 2018	981 (40.9)	1.52 (1.15–1.99)	1.44 (1.09–1.90)
July 2018 to December 2018	487 (20.3)	2.42 (1.80–3.24)	1.78 (1.30–2.44)
Referral source			
VCT (reference)	1,844 (76.9)		
PMTCT	262 (10.9)	0.81 (0.54–1.20)	0.76 (0.50–1.14)
Missing	291 (12.1)	0.71 (0.48–1.04)	0.59 (0.40–0.88)
BMI			
<18.5	267 (11.1)	0.93 (0.59–1.45)	
18.5–24	1,147 (47.8)	1.26 (0.93–1.70)	
>24 (reference)	512 (21.4)		
Missing	471 (19.7)	1.17 (0.82–1.67)	
WHO stage			
Stage I (reference)	1,572 (65.6)		
Stage II	288 (12.0)	0.80 (0.55–1.17)	
Stage III and IV	121 (5.1)	0.54 (0.28–1.06)	
Missing	416 (17.4)	1.03 (0.77–1.38)	

aHR, adjusted hazards ratio; CI, confidence interval; HR, hazards ratio.

Among 2,524 patients, 1,698 (67.3%) had available data on viral load within 455 days after enrollment in care. Of these, 1,476 (86.9%) were virally suppressed. A higher proportion of patients initiating ART on the same day as enrollment (89%) were virally suppressed compared with those initiating ART 1–7 days (84%) or >7 days (88%) after enrollment (*p* = .04). In multivariable logistic regression, this association was not statistically significant (Table 3). Underweight patients (BMI <18.5 kg/m^2^), compared with those with BMI >24, had half the odds of being virally suppressed (aOR = 0.56 and 95% CI = 0.33–0.95).

**Table 3.** Associations of Demographic and Clinical Characteristics at Enrollment and Viral Load Suppression Among Patients with Available Viral Load Data (*N* = 1,698)

Variable	VL <200,* n *(%)	VL ≥200,* n *(%)	OR (95% CI)	aOR (95% CI)
Time to ART initiation				
Same day (reference)	505 (89.1)	62 (10.9)		
1–7 days	489 (84.2)	92 (15.8)	0.65 (0.46–0.92)	0.73 (0.50–1.04)
>7 days	482 (87.6)	68 (12.4)	0.87 (0.60–1.26)	1.01 (0.69–1.48)
Sex				
Female (reference)	855 (88.8)	108 (11.2)		
Male	621 (84.5)	114 (15.5)	0.69 (0.52–0.91)	0.78 (0.58–1.05)
Age (years)				
15–23	154 (85.6)	26 (14.4)	0.96 (0.60–1.53)	
24–35	753 (87.9)	104 (12.1)	1.17 (0.87–1.58)	
>35 (reference)	569 (86.1)	92 (13.9)		
CD4 count (cells/mm^3^)				
≥500 (reference)	376 (88.7)	48 (11.3)		
350–499	229 (88.4)	30 (11.6)	0.97 (0.60–1.58)	1.05 (0.64–1.71)
200–349	233 (87.9)	32 (12.1)	0.93 (0.58–1.50)	1.08 (0.66–1.75)
<200	165 (78.9)	44 (21.1)	0.48 (0.31–0.75)	0.57 (0.36–0.90)
Missing	473 (87.4)	68 (12.6)	0.89 (0.60–1.32)	0.94 (0.63–1.41)
Enrollment period				
July 2016 to June 2017 (reference)	579 (85.7)	97 (14.3)		
July 2017 to June 2018	615 (87.1)	91 (12.9)	1.13 (0.83–1.54)	
July 2018 to December 2018	282 (89.2)	34 (10.8)	1.39 (0.92–2.11)	
Referral source				
VCT (reference)	1,143 (86.2)	183 (13.8)		
PMTCT	166 (91.2)	16 (8.8)	1.66 (0.97–2.84)	
Missing	167 (87.9)	23 (12.1)	1.16 (0.73–1.85)	
BMI (kg/m^2^)				
<18.5	173 (83.2)	35 (16.8)	0.47 (0.28–0.78)	0.56 (0.33–0.95)
18.5–24	691 (85.2)	120 (14.8)	0.55 (0.36–0.82)	0.58 (0.38–0.88)
>24 (reference)	347 (91.3)	33 (8.7)		
Missing	265 (88.6)	34 (11.4)	0.74 (0.45–1.23)	0.68 (0.38–1.22)
WHO stage				
Stage I (reference)	994 (87.7)	139 (12.3)		
Stage II	176 (81.5)	40 (18.5)	0.62 (0.42–0.91)	0.70 (0.47–1.05)
Stage III and IV	73 (83.9)	14 (16.1)	0.73 (0.40–1.33)	0.83 (0.45–1.55)
Missing	233 (88.9)	29 (11.1)	1.12 (0.73–1.72)	1.05 (0.63–1.76)

OR, odds ratio.

## Discussion

In this analysis of data prospectively collected from patient medical files at 10 health centers in Kigali, Rwanda, we found that patients who initiated ART on the same day as enrollment were more likely to be lost to care compared with those who initiated ART >7 days after enrollment. However, we found no statistical differences between those initiating ART 1–7 and >7 days after enrollment, suggesting that same-day ART may lead to higher loss to care among Rwandan PLHIV initiating ART under Treat All.

These findings are consistent with several studies also showing that same-day ART initiation was associated with loss to care.^7–9^ Studies that compared rapid ART initiation with standard of care (ART initiation after 1 or 2 weeks after diagnosis) found improved retention in care and clinical outcomes^19,20^ contrary to studies comparing same-day ART initiation and one or more days as previously described. This indicates that same-day ART initiation may be the driver of the observed loss to care. Our study is among the first to examine the differences between same-day ART initiation and ART initiation 1–7 days after diagnosis.

In addition, there are concerns raised by health care providers and some policy makers that ART initiation quickly after diagnosis may lead to disengagement from care if patients have not accepted their diagnosis and are not prepared for ART initiation.^21–23^ Engagement in care may be negatively impacted by stigma that continues to surround HIV infection as indicated by a study carried out in Rwanda where newly diagnosed patients reported that taking medication and attending appointments were stigmatizing.^24^ Same-day ART initiation in some settings has had a negative impact on retention in care, suggesting a need for ART programs to weigh carefully the benefits versus risks of same-day ART initiation. To improve outcomes, early ART initiation can be offered together with some interventions aimed at improving retention in care, such as additional counseling even by lay counselors and peers, differentiated service delivery models and financial incentives.^25^

We did not find a statistically significant difference in viral suppression between those who initiated ART on the same day as diagnosis and those who initiated it 1–7 and >7 days after diagnosis. Although same-day ART initiators had a higher proportion of viral suppression compared with other groups, the lack of a statistically significant association suggests that waiting for a day or two or for the patient to get ready might not negatively impact viral suppression among those retained in care. This has been shown by several studies indicating that early or rapid ART initiation is associated with improved viral suppression.^20,26,27^ We found low BMI to be associated with lower odds of viral suppression; this may be explained by more advanced disease among this group, as we observed a trend for underweight patients to have lower CD4 counts (data not presented).

We found that younger patients (those aged 15–24 years) were more likely to be lost to care than those aged >24 years, a finding that is consistent with recent data from the Rwanda Population-based HIV Impact Assessment^12^ as well as many prior studies conducted in SSA.^28–30^ With increasing HIV infections among young people and them not succeeding under Treat All, further efforts should be made to improve those poor outcomes to ensure that the benefits of Treat All reach all. Our finding that enrolling between July and December 2018 was associated with loss to care may be owing to a couple of factors. One possibility is that, as Treat All was rolled out in Rwanda and as more and more patients were prescribed ART rapidly after diagnosis, counseling and close follow-up of patients in their early phases of HIV care became limited and retention in care was negatively impacted. Alternatively, although we only included participants who had at least 1 year of follow-up time, it is possible that these results reflect a lag in data availability for the July 2018 to December 2018 period.

Our study has several limitations related to the use of routine, observational data. First, although WHO guidelines recommend ART initiation within 7 days of HIV diagnosis, we could not definitively ascertain HIV diagnosis date and therefore were limited to examining time from HIV care enrollment to ART initiation. We were not able to measure other important factors that may influence decision of time to ART, such as readiness to begin ART, presence of opportunistic infections, or adequate counseling. We also did not include patients who had transferred from other facilities, possibly impacting external validity of our findings. In addition, we were unable to ascertain outcomes among patients lost to care, which include unascertained deaths and silent transfers. Patients lost to care may still be alive and on ART.^31^ Additional research efforts by our team are underway to understand outcomes among patients who do not return for appointments. The relatively small subsample and the limited number of loss to care events may have resulted in less precise estimates of loss to care. Finally, this was a largely urban cohort in a country with a relatively low HIV prevalence. Therefore, our findings may not be fully generalizable to other settings.

## Conclusions

In summary, we found that patients in Rwanda initiating ART on the same day as enrollment in HIV care were more likely to be lost to care than those initiating later. There is increasing evidence for the benefit of rapid (including same day) ART initiation but concerns remain about patients starting ART before they are ready,^32^ which may adversely impact adherence and treatment outcomes. Our findings, as well as those from other studies, suggest that ensuring adequate, ongoing support for PLHIV initiating ART rapidly is important to maintain engagement in care and ultimately achieve treatment success for newly diagnosed PLHIV in the era of Treat All. Finally, there is need to further investigate the reasons for loss to care so that appropriate interventions can be designed and targeted.
